# The intercultural development and validation of the Indigenous ‘Escala de Bienestar Kankuamo’ (Kankuamo Well-Being Scale): A capability approach

**DOI:** 10.1177/13634615251359777

**Published:** 2025-08-17

**Authors:** C. F. Van der Boor, D. M. Agudelo-Ortiz, G. C. Sánchez Díaz, C. I. Molina-Bulla, L. J. Guevara Morales, A. Chiumento, D. M. Aponte Canencio, R. White

**Affiliations:** 1 Department for Health Services Research and Policy, London School of Hygiene and Tropical Medicine, London; 2 Faculty of Social and Human Sciences, Externado University of Colombia, Bogotá; 3 School of Social and Political Science, University of Edinburgh, Edinburgh; 4 1596 School of Psychology, Queen's University Belfast, Belfast

**Keywords:** Indigenous, well-being scale, capability approach, mixed-methods, participatory research

## Abstract

This article describes the development and validation of a capability-based well-being scale: ‘Escala de Bienestar Kankuamo’ (EBK; the Kankuamo Well-Being Scale). The EBK is designed to measure the well-being of the Indigenous Kankuamo community of Colombia from an intercultural perspective. The mixed-methods study was composed of two phases. In phase I, an initial 27 items, which had been generated using qualitative data from a previous study, were reviewed and adjusted through workshops with the Kankuamo community. In phase II, an adapted list of 28 items was piloted within the Kankuamo communities (sample *N* = 213). A preliminary exploratory factor analysis was carried out. The internal consistency of the EBK was measured using McDonald's ω. Convergent and divergent validity were tested with the World Health Organization-Five Well-Being Index (WHO-5) and the Patient Health Questionnaire-9. Incremental validity was tested through a hierarchical regression analysis to determine the effect on the WHO-5 of age, gender, community and EBK. The exploratory factor analysis resulted in a 14-item scale with seven domains: (1) Relating (to others and one's emotions), (2) Guiding principles, (3) Choice of healthcare, (4) Self-grown food, (5) Community contributions, (6) Agency and (7) Respect and equality. The EBK had high internal consistency (McDonald's ω = 0.86). Statistical analyses aimed at determining the convergent and divergent validity were inconclusive, suggesting a need for caution in using western standardised mental health measures in Indigenous communities because these may lack adequate cultural fit. The findings of the current study identify local priorities and needs, and evidence the feasibility of operationalising the capability approach for Indigenous populations.

## Introduction

Colombia is home to more than 1.9 million Indigenous people (Departamento Administrativo Nacional de Estadística, 2020). Because of violence and internal displacement, they are considered one of Colombia's most vulnerable groups (United Nations High Commissioner for Refugees, 2012). Indigenous people experience higher mortality and morbidity rates than the general population (MINSALUD, 2016), and have elevated rates of depression, anxiety (Gómez-Restrepo et al., 2015), substance abuse (Arévalo et al., 2013) and suicide (Valle & Jiménez, 2012).

The dominance of a biomedical model of health within the Colombian national healthcare system has been highlighted as an important issue for Indigenous people (Caballero Pineda, 2019). Indigenous communities have developed knowledge systems in which a complex relationship exists between biological and ecological resources, conceptions of life and history of communities, as well as between the environment, living beings, ancestors and spirits (Aponte-Canencio & Agudelo-Ortiz, 2016; Patiño Suaza & Sandín Vásquez, 2014). No separation is made between biological and cultural elements of the self (Portela-Guarín, 2014), nor the physical, psychological and spiritual dimensions of health (Agudelo-Ortiz, 2013). Colombia is working on an Intercultural Indigenous Health System, aiming to respond to the quest of over 102 Indigenous communities to gain the right to high quality healthcare that recognises and values their traditional knowledges (Aponte-Canencio & Agudelo-Ortiz, 2016). This is in line with the United Nation’s call for health systems and measures that are appropriate to Indigenous contexts (United Nations, 2018). This process calls for the use of health indicators that are sensitive to ancestral health knowledges. This article describes the co-development and validation of a novel Indigenous well-being scale with the Indigenous Kankuamo peoples of Colombia, applying the capability approach (CA; Sen, 1999).

### The capability approach and Indigenous peoples

The CA is a human development paradigm that puts people at the centre of initiatives aimed at improving their lives. The CA states that what truly matters for well-being and development is whether people have opportunities to engage in forms of doing and being they have reason to value. This is described as comprised of functionings – the valuable activities and states that make up people's well-being (being healthy) – and capabilities – the substantive freedoms an individual has to lead the life they have reason to value (access to food; Sen, 1999). Gigler (2005) highlights the overlaps between Indigenous worldviews and the CA, in that both take ‘a holistic view … and stress the multiple dimensions of development as the expansion of people's well-being including social, cultural and spiritual elements’ (p. 1). Applying the CA to support the development of Indigenous communities provides opportunities to shift understandings of well-being away from an over-emphasis on material conditions as defining development, towards greater recognition of the value that traditional knowledge and cultural identity provide (Gigler, 2005).

Efforts to apply the CA in research with Indigenous communities provides opportunities for shared intercultural understandings of how well-being is conceptualised, and to deliver outcomes that are meaningful and attainable in Indigenous contexts (Entwistle & Watt, 2013). Sen also advocates for the use of open, participatory approaches to understanding well-being and for ensuring that researchers’ values are not (un)intentionally imposed (Sen, 1989). Effective participatory research must treat people as moral equals, ensure fairness in the research processes, and take historical and cultural contexts seriously (Nuffield Council on Bioethics, 2020).

The principles of the CA share common features with those of tribally based participatory research (TBPR; Fisher & Ball, 2003). TBPR offers an ethical and methodological stance that prioritises self-determination and the empowerment of Indigenous communities to actively steer and guide research. The TBPR methodology is characterised by tribal governments and communities taking the lead in research or exercising agency in extending invitations to collaborators. Our study sought to uphold principles aligned with the ethical values delineated by both the CA and TBPR. For example, the involvement of international collaborators in this research was driven by invitations from the Kankuamo communities to pursue the health and well-being goals they had defined as priorities.

### The Indigenous Kankuamo people

The Kankuamo inhabit the Sierra Nevada de Santa Marta, in northern Colombia. They refer to this region as ‘the heart of the world’ and live by the mandate of maintaining their ancestral territory as a sacred place for the planet (Resguardo Indígena Kankuamo, 2010). The 2020 national census of Colombia estimated that there are 25,000 Kankuamo people, of whom 14,000 live within this ancestral territory across 12 communitiesTS.^1^ The Kankuamo have been exposed to conquest, colonialism, forced displacement, labour exploitation, sexual abuse, illegal extraction of natural resources from within their territories, forced conversion to Christianity, and other forms of structural violence and oppression that threaten their tradition and culture (Farmer, 2004; Farmer et al., 2006; Office of the United Nations High Commissioner for Human Rights, 2025). Over the past decade, the Kankuamo have been advancing processes of collective reparations and memory-building with the support of state entities and local Indigenous organisations (Cabildo Indígena del Resguardo Kankuamo, 2021; Centro National de Memoria Histórica, 2019). However, they face ongoing threats, for example due to infrastructure projects (such as hydroelectric dams), that disrupt their traditional and spiritual way of life. Meanwhile, a history of prolonged displacement has led to a merging of traditional knowledges with dominant biomedical systems and knowledges in the field of healthcare.

In response to this historical and structural context, the Kankuamo are seeking to consolidate a healthcare model that integrates their worldview with the biomedical model prevalent in Colombia. This is being pursued through the recovery of their identity, ancestral knowledge and practices. In this, health is defined through a spiritual, ancestral interpretation and in relation to the Law of Origin, which is understood as a relationship of harmony between all beings, people, nature and spirits within the territory (Kankuama, 2014). As part of this process, the Kankuamo peoples are working on the participatory construction of an intercultural epidemiological profile.^2^ This article describes the development and validation of an assessment instrument called the ‘Escala de Bienestar Kankuamo’ (EBK; in English the ‘Kankuamo Well-Being Scale’), which was designed to measure the well-being of the Kankuamo people from a CA-based and intercultural perspective. In line with underpinning principles of the CA and TBPR, the Kankuamo community had comprehensive decision-making authority over all research activities described in this article.

## Methods

A two-phased mixed-methods approach was used. Prior to the current study, preliminary items were developed in a qualitative study aiming, as part of a broader project, to support the recovery of identity and spiritual knowledge of the Kankuamo people. This study (Boor et al., 2024) investigated what a ‘good life’ means to the Kankuamo people through focus groups discussions. The Spanish wording was extracted verbatim from transcripts and used to create an initial draft of EBK items. In phase I of the current study, preliminary items were refined through a Kankuamo stakeholder workshop (*n* = 24). These workshop participants, who were all members of the community, were then invited to become members of the research team in phase II in which the refined version of the EBK was piloted with a larger sample of community members. Ethical approval was granted by the research ethics committee of the Externado University of Colombia (Concept No.40) and acknowledged by the Faculty of Engineering and Physical Sciences Research Ethics Committee of Queen's University of Belfast (Reference: EPS 21_394).

### Striving to attain equitable research relationships

Efforts were made to attain principles of equitable research relationships. The relationship between the Colombian academic research team (D.M.A., G.C.S.D., C.I.M.B., L.J.G.M., D.M.A.C.) and the Kankuamo community was established three years prior to the project grant application. This was initiated by the Kankuamo community's request for collaboration to support recuperation of their traditional knowledges through intercultural dialogue. To attain these goals, Colombian partners, with the support of the Kankuamo community, built further relationships with the UK-based research team, and co-developed a successful funding application to carry out this study.

The stance of the academic researchers involved in this study is one of horizontal and mutual learning, which seeks to embrace the complexities of the interculturality shaping research encounters, and is embedded in a relational dynamic grounded in an ethics of care as well as ontological and epistemological humility. Awareness of intercultural complexity entails recognising the power and privilege that are associated with the UK partner holding and administering the grant budget, and being reflexive about the implications of white, European individuals being invited into Kankuamo territories as academic researchers bringing methodological ‘expertise’. In relation to this article, expertise in the development and testing of assessment scales was contributed by C.v.d.B. and R.W., supported by other co-authors.

A core aim of the partnership was to build capacity in the development and application of well-being scales, thereby empowering the community to respond to their own needs in the future. This was achieved through the integration of Colombian collaborators and local community members into research teams and by providing training and support in the qualitative and quantitative methods described in this article. Efforts were made to address the inequities that, inevitably, remain present in this structural context. The Kankuama IPS, which is the Indigenous-focused health service provider within the Colombian health system, retained decision-making authority as community representatives. In addition, all research activity was jointly developed and conducted in partnership with Kankuamo community representatives and members. The latter did not wish to be included as authors of this academic paper, requesting instead to have their contributions identified in the Acknowledgements.

### Phase I: Refinement of the EBK

An initial pool of 27 items derived from the previous qualitative study was reviewed and refined for content and face validity through a face-to-face workshop in Spanish, the primary language spoken by the Kankuamo communities. The need to involve local communities in item development has previously been emphasised for content validity (Rubio et al., 2003; Vogt et al., 2004). Workshop participants (*n* = 24) responded to an invitation to Kankuamo representatives. Eighteen were female and six were male, with a mean age of 34 (range 21–62) years. It is also important to note that most workshop participants came from larger Kankuamo communities. Recruitment was purposive, targeting individuals who had been participating in a one-year programme on intercultural health within the Indigenous reserve^3^. This programme aimed to create a legacy of traditional Kankuamo knowledge. The EBK workshop was embedded within this programme to provide basic knowledge on instrument development and the conduct of health surveys. Participation was voluntary, and transport and food costs were covered. As part of the intercultural health programme, participants had previously provided written consent.

Workshop participants were divided into three groups to review the 27 items. Each group was facilitated and supported by a member of the research team. The groups were asked to discuss three questions for each item: (a) Is this item clearly expressed in the local language? If not, how should it be amended? (b) Is it acceptable to ask this question in the local context, or could some participants feel uncomfortable answering this question honestly? If it is not acceptable, how should it be amended? (c) Is this item relevant in the local context? The third question was answered on a 5-point Likert scale (1, strongly agree to 5, strongly disagree). These questions are consistent with the ‘cognitive interviewing techniques’ used in the adaptation and development of assessment instruments (Christodoulou et al., 2008). The groups were asked four additional questions: (a) Do the items adequately reflect the dimensions of well-being for the Kankuamo people? (b) How do you feel listening to the questions on the EBK? (c) Are there any dimensions of well-being that have not been captured in the EBK? (d) Is the response format adequate? The three researchers took written notes of each group's discussion. After initial responses, suggested amendments were discussed with the whole group. As a result, two items were deleted, eight were amended and three were added. Twenty-eight items were retained in the EBK V.01, each of which is answered on a 5-point Likert scale (0, strongly disagree to 4, strongly agree). For the original measure see Appendix A.

### Phase II: Factor analysis and validation

In phase II, the EBK was piloted in a community sample. An exploratory factor analysis (EFA) and preliminary investigation of the validity and internal consistency were conducted to determine whether the EBK is a psychometrically sound measure of capabilities in the Kankuamo community. A paper-based cross-sectional design was used to then conduct a community survey that included the EBK. Data was collected between 22 January and 2 February 2022. In line with participatory action research, phase I participants who were willing joined the research team for phase II. They were trained to act as community assessors who carried out the consent procedures and went through the EBK with participants. Each community assessor carried out between five and ten assessments within their own community. A total of 35 community assessors participated, including 24 individuals who came to the workshops (phase I), and 9 who were enrolled in the intercultural health programme but were unable to attend the workshops.

The community survey recruited a convenience sample. Eligibility criteria were being adult (>18 years), self-identifying as Kankuamo, and having the capacity to provide informed consent. Written informed consent was requested from each participant prior to participating. Three participants were illiterate and provided a fingerprint instead. Consent was witnessed for all included participants. Seven participants were excluded because of the absence of a witness. The final sample consisted of 213 participants, living in 11 different communities (Table 1). Participants did not receive compensation for their time.

**Table 1.** Participant sociodemographic information (*N* = 213).

Characteristic	*n* (%)
Community	
Atanquez	55 (26)
Ramalito	27 (13)
Pontón	21 (10)
Chemesquemena	20 (9)
Rancho de la Goya	18 (9)
Guatapurí	18 (9)
Los Haticos	13 (6)
La Mina	11 (5)
Mojao	11 (5)
Murillo	11 (5)
Valledupar	7 (3)
Missing	2 (1)
Gender	
Male	100 (47)
Female	112 (53)
Other	1 (0)
Age	*M* = 37, *SD* = 15
Born in Indigenous territory	
Yes	178 (84)
No	29 (14)
Unsure	3 (1)
Years living in Indigenous territory (years)	*M* = 32, Range = 1–81
Indigenous education	
Yes	157 (74)
No	49 (23)
Government education achieved	
Primary school	174 (82)
Secondary school	142 (67)
Accessed university or technical training	64 (30)
Civil status	
Single	71 (33)
Married	16 (8)
Separated	9 (4)
Divorced	0
Widow	8 (4)
Free union	108 (51)
Other	1 (0)
Employment	
Salaried	33 (15)
Self employed	196 (46)
Unpaid employment	3 (0)
Student	80 (9)
Head of household	190 (18)
Retired	6 (0)
Unemployed, health reason	35 (2)
Unemployed, other reason	72 (4)
Other	72 (4)
Children	
Yes	154 (72)
No	58 (27)
Number of children	*M* = 3.46

This study used two further measures to assess the convergent and divergent validity of the EBK. No CA-based measures are currently available in Spanish that have been validated in an Indigenous context. Therefore, we instead used globally recognised measures of well-being (World Health Organization-Five Well-Being Index; WHO-5) and depression (Patient Health Questionnaire-9; PHQ-9) that have previously been used in Indigenous settings (e.g., Faruk et al., 2021; Williams et al., 2018).

The PHQ-9 (Kroenke et al., 2001*)* is a short self-report measure of depression. It is composed of nine items, with a score ranging from 0 (not at all) to 3 (nearly every day). A threshold score of 10 or higher is considered to indicate mild depression, a score of 15 or higher moderate depression, and a score of 20 is indicative of severe major depression. It has been translated and validated in Spanish (Baader et al., 2012; Cassiani-Miranda et al., 2021). An example item is ‘Tener poco interés o placer en hacer las cosas’ (Little interest or pleasure in doing things). The PHQ-9 has demonstrated high internal consistency (McDonald ω = 0.83).

The *WHO-5* ( WHO, 1998*)* is a short well-being measure developed from items of the Zung scale for depression, distress and anxiety (Zung, 1965), the General Health Questionnaire (Goldberg & Williams, 2000), and the Psychological General Wellbeing Scale (Dupuy, 1984). It is composed of five items rated on a six-point Likert scale (5, all the time to 0, at no time), and has been officially translated into Spanish. An example item is ‘Me he sentido active y enérgico’ (I have felt active and vigorous). The WHO-5 has demonstrated high internal consistency (McDonald’s ω = 0.81).

An expectation maximisation analysis was carried out for participants who had missing data for a maximum of two answers on the EBK (*n* = 8) and the PHQ-9 (*n* = 8). The WHO-5 had no missing data. The EBK data was ordinal, scored on a five-point Likert scale, therefore a parallel analysis was conducted using a simulated polychoric correlation matrix to identify the number of likely components in the data. Following the parallel analysis, an EFA was carried out on the polychoric matrix to understand the underlying factor structure. An oblique rotation was applied because factors were expected to be correlated (Fabrigar et al., 1999). Items with a factor loading of .35 or higher were retained.

The internal consistency was estimated by computing McDonald's ω. To test convergent validity, the EBK and the WHO-5 were correlated, because both intend to measure well-being. For the divergent validity, the EBK and PHQ-9 were correlated because they intend to measure opposite constructs (depression and well-being). Lastly, incremental validity was tested using a hierarchical regression to assess the effect on levels of well-being (WHO-5) of age, gender, community and EBK. All analyses were run in RStudio version 4.1.3.

## Results

### Factor structure

Parallel analysis of the 28-item EBK (v.0.1) suggested there were up to seven underlying factors; seven was therefore set as an upper limit when exploring EBK structure using EFA. The Kaiser–Meyer–Olkin measure (KMO) suggested good sampling (0.84) and Bartlett's test of sphericity was significant, indicating that correlations between items were large enough to carry out an EFA (*χ*^2^ (378) = 1131.663, *p* < .001).

Eigenvalues for the seven factors were as follwos: factor 1 = 2.80 (variance explained = 10%); factor 2 = 2.45 (variance explained = 9%); factor 3 = 1.94 (variance explained = 7%); factor 4 = 1.47 (variance explained = 5%); factor 5 = 2.88 (variance explained = 10%); factor 6 = 2.69 (variance explained = 10%); and factor 7 = 2.29 (variance explained = 8%). Based on this initial analysis, a seven-factor solution was retained.

Using a cut-off value of 0.40, three items loaded onto factor 1, and one item loaded onto factors 2, 3 and 4. Three items loaded onto each of factors 5 and 6, and two items loaded onto factor 7. A factor with fewer than three items is generally considered weak and unstable. However, given the relevance to the Kankuamo community of the single items on factors 2, 3 and 4, and following community consultation, it was agreed to retain these as single items on the measure. The resulting 14-item EBK (v.1.0) demonstrated good internal consistency (McDonald's ω = 0.86), and each of the factors constituted a meaningful domain: (1) Relating (to others and one's emotions), (2) Guiding principles, (3) Choice of healthcare, (4) Self-grown food, (5) Community contributions, (6) Personal agency and (7) Respect and equality. Each factor also demonstrated good internal consistency (Table 2).

**Table 2.** Factor structure of the Escala de Bienestar Kankuamo (EBK; version 1.0). Scale items are provided in Spanish and English.

		Rotated factors^1^						
	Scale item	Factor 1	Factor 2	Factor 3	Factor 4	Factor 5	Factor 6	Factor 7
1.	Soy capaz de mantener buenas relaciones con las personas que son importantes para mi (I am able to maintain good relationships with the people that are important to me)	.**63**	.11	.01	.18	−.03	.12	.13
2.	Logro contribuir a la armonía en mi hogar (I am able to contribute to the harmony within my home)	.**75**	.02	.07	−.01	.16	.05	.05
3.	Soy capaz de comportarme de acuerdo a las situaciones que se presentan (I am able align my behaviour to different situations that occur)	.**59**	.01	.20	−.23	.11	.12	.15
4.	Tengo la oportunidad de orientar mis acciones a través de los principios de la Ley de Origen o mis propias creencias (I have the opportunity to guide my actions through the principles of the Law of Origin or my own beliefs)	.03	.**72**	.05	.05	.05	.04	−.17
5.	Tengo la oportunidad de decidir entre usar medicina tradicional y occidental (I have the opportunity to decide between using traditional and western medicine)	.02	0	.**9**	.01	.03	.02	.03
6.	Tengo oportunidad de producir (cultivar, criar) mis propios alimentos (I have the opportunity to produce (grow, cultivate) my own food)	−.12	.01	.06	.**64**	.18	.04	0
7.	Dentro de mis posibilidades, puedo contribuir a las actividades colectivas de la comunidad (Within my possibilities, I can contribute to the collective activities of the community)	−.02	−.09	.07	.18	.**71**	.03	.13
8.	Soy capaz de cumplir con las funciones que tengo dentro de la comunidad (I am able to fulfil the functions that I have within the community)	.13	0	.03	0	.**84**	.03	−.09
9.	Tengo la oportunidad de discutir mis intereses con las autoridades de mi comunidad (I have the opportunity to discuss my interests with the authorities of my community)	.02	.29	−.12	−.04	.**56**	−.05	.24
10.	Tengo la libertad de tomar decisiones personales que son importantes para mi (I have the freedom to make personal decisions that are important to me)	.2	.02	.07	.03	−.10	.**64**	.18
11.	Puedo expresar mi opinión libremente (I am able to express my opinions freely)	.24	−.02	.16	.04	.1	.**47**	.03
12.	Puedo participar en actividades culturales y deportivas que son importantes para mi (I am able to participate in cultural and sport activities that are important to me)	−.11	−.06	.06	.05	.17	.**63**	−.11
13.	Dentro de mi territorio, me tratan con el mismo respeto y consideración, independientemente de mi género, etnia, edad, estado civil, etc. (Within my territory, I am treated with the same respect and consideration, regardless of my gender, ethnicity, age, relationship status, etc.)	.29	−.03	.14	−.10	.29	−.04	.**56**
14.	Soy capaz de reconocer y respetar a las personas por igual, independientemente de su género, etnia, edad, estado civil, etc. (I am able to recognise and respect people equally, regardless of their gender, ethnicity, age, relationship status, etc.)	.09	−.21	.12	.14	−.03	.20	.**57**

*All significant loadings are given in bold.

^1^ Rotated factors: Factor 1, Relacionarse (con los demás y las emociones de uno)/Relating (to others and one's emotions); Factor 2, Principios Guía/Guiding principles; Factor 3, Elección de Atención Médica/Choice of healthcare; Factor 4, Cultivo propio/Self-grown food; Factor 5, Contribuciones Comunitarias/Community contributions; Factor 6, Agencia/Agency; Factor 7: Respeto e Igualdad/Respect and equality.

### Convergent and divergent validity

The convergent validity of the EBK was tested using the WHO-5. Overall EBK scores were not significantly correlated with the WHO-5 measure (*r*_s_(211) = .07, *p* = .309). Nor were any of the subscales correlated with the WHO-5. The divergent validity of the EBK was tested using the PHQ-9. The overall EBK scores were also not significantly correlated with the PHQ-9 (*r*_s_(211) = .03, *p* = .673). Subscale 4 (Self-grown food) was significantly correlated with the PHQ-9 (*r*_s_(211) = .19, *p* = .005; Table 3).

**Table 3.** Mean Scores and Spearmans Correlations Between Each of the Scales and Subscales.

Variable	*M* (*SD*)	1.	2.	3.	4.	5.	6.	7.	8.	9.	10.
1. World Health Organization-Five Well-Being Index	20.35 (4.26)										
2. Patient Health Questionnaire-9	3.74 (3.88)	−.43**									
3. Escala de Bienestar Kankuamo	44.61 (5.11)	.07	.03								
4. Subscale 1: Relating (to others and one's emotions)	9.86 (1.43)	.08	−.04	.74**							
5. Subscale 2: Guiding Principles	2.87 (0.84)	.09	−.02	.45**	.20*						
6. Subscale 3: Choice of Healthcare	3.30 (0.55)	−.07	.11	.47**	.34**	.19*					
7. Subscale 4: Self-grown Food	3.31 (0.82)	−.03	.19*	.42**	.14*	.15*	.21*				
8. Subscale 5: Community Contributions	8.98 (1.92)	.12	−.02	.76**	.39**	.32**	.26**	.29**			
9. Subscale 6: Agency	9.79 (1.38)	.02	.04	.70**	.49**	.21*	.31**	.23**	.37**		
10. Subscale 7: Respect and Equality	6.49 (1.01)	−.02	.03	.69**	.65**	.07	.36**	.15*	.41**	.46**	

*p < .05, **p < .001.

Given the lack of significant findings of convergent and divergent validity, we considered it necessary to undertake a community validation exercise. The Kankuamo have oral knowledge-sharing traditions, in which community members practice daily community rituals to share ideas and reflect in a group setting. We brought the EBK items to one of these groups for validation from the community. To avoid social bias, the academic researchers were not involved. Members from the community orally reviewed the items and subscales, and then provided feedback to the research team. We learned that community members approved of the items but considered it necessary to include an additional ranking exercise, wherein respondents are asked to rank the subscales in order of importance to them. They thought that individuals may prioritise domains differently, and that their preferences should be reflected. The requested ranking exercise is currently underway and findings will be published in the EBK guidelines developed for the community. This request confirms the importance of intercultural validation, not only for the development of the EBK, but also more broadly in CA-based research. In the final EBK, the total score (ranging from 0 to 56) should be reported along with the rank order of subscales.

### Hierarchical regression

To test the incremental validity of the EBK, a hierarchical regression was run to analyse the effects of age, gender, community and EBK score on levels of well-being (WHO-5). Age, gender and community were entered at step 1, and the EBK score at step 2. Variance inflation factors suggested no multicollinearity. The final regression model was significant and explained 5.1% variance (*F*(4, 207) = 2.80 p = .027). Including the EBK score accounted for an additional 0.6% variance. Age and community were not significant predictors of well-being, but gender was (*β* = −1.75, *p* = .002), with males scoring higher. The EBK measure was not a significant predictor (*β* = 0.07, *p* = .245) (Table 4).

**Table 4.** Hierarchical regression to test the effects of age, gender, community and the Escala de Bienestar Kankuamo (EBK) score on levels of well-being (WHO-5).

Variable	Cumulative		Simultaneous	
	*R*^2^-change	*F*-Change	*β*	*p*
Step 1	.05	3.28*		
Age			.00	.808
Gender			−1.75	.002*
Community			.07	.498
Step 2	.01	2.80		
EBK			.07	.245

*p < .05.

## Discussion

There have been calls for the development of culturally congruent health measures, interventions and systems to decolonise healthcare and promote the well-being of Indigenous populations (Dickerson et al., 2020). We sought to contribute to this effort through the intercultural coproduction and validation of the EBK; an instrument designed to measure the well-being of the Kankuamo Indigenous people of Colombia. The CA was central to the development of the EBK. Sen (1990) argues that what matters is what people are capable of being or doing with the goods and resources they have access to. The CA provides a broader evaluation space for conceptualising development and well-being than more traditional western approaches that typically focus on resources and/or utility (Nussbaum, 2000; Sen, 1983, 1999). This aligns with Indigenous perspectives, because the CA recognises the different ways in which lives can be enhanced or impoverished beyond income, wealth, resources or primary goods. Indeed, the EBK showcases that the Kankuamo people have broad perspectives on well-being that prioritise its non-monetary dimensions, and emphasise the role of traditional epistemologies such as the Law of Origin. The participatory process of refining and validating the EBK illustrates how the CA can be applied in practice; by centring the knowledge, values and priorities of the community, it affirms their agency in defining what well-being means in their own context. In doing so, the study not only aligns with the ethos of the CA, but also demonstrates its usefulness in operationalising well-being in Indigenous settings.

The framing of CA-based understandings of well-being that emerged in this study is reflected in other CA-based indicators of well-being developed in diverse Indigenous settings, while also highlighting context-specific differences (Acosta et al., 2020; Tellez Cabrera, 2022; Yap & Yu, 2016). For example, the CA-based indicators of well-being identified by Yap and Yu (2016) in a study with the Yawuru aboriginal community in Australia highlighted themes of family: identity and relatedness, community, safety and respect, connection to culture, rights and recognition, and health. These are similar to the themes arising in the development and validation of the EBK. By contrast, they identified themes of standard of living and connection to country that were not replicated in the current study (Yap & Yu, 2016). In addition, the Subjective health Capabilities in Adults tool (CAPSAS_a) developed with the Purépecha community in Mexico included 11 indicators of well-being distributed across four dimensions of: (a) health – including physical, mental and social indicators; (b) health agency – including indicators on preventive measures to protect health, health-goal achievement, knowledge of traditional medicine and access to health-related information; (c) access to health services – including indicators on availability of providers, medication and tests; and (d) community – with indicators on healthy lifestyles and safety (Cabrera 2021). Although many of these indicators overlapped with those found in the current study, there are notable differences. For instance, the EBK included additional indicators on guiding principles, self-grown food and, to some extent, other forms of agency. These sets of indicators highlight that although some capabilities might be transferrable across settings, such as health agency, relatedness, safety and respect, others might be more context specific (self-grown food). These findings are commensurate with Sen's views on the CA as a theory and method for identifying and working to attain contextually bound capabilities relevant to the goods and resources available in particular communities (Stiglitz et al., 2009). Evidence of holistic approaches to well-being invites the question of how local mental health services can better speak to these context-specific social and cultural needs. Furthermore, it suggests that outside agencies and state actors need to consider providing support beyond financial and material resources, as these may not be sufficient to expand capabilities and increase well-being.

The current study found that the EBK's convergent and divergent validity were non-significant. These findings can be interpreted in different ways. First, this could mean that the EBK is not a valid measure of capability-based well-being within the Kankuamo community. We consider this unlikely, given that this tool was developed using qualitative data collected within the community and received multiple rounds of feedback from experts with lived experience. A second interpretation is that neither the WHO-5 nor the PHQ-9 is an appropriate instrument for investigating the validity of a novel measure of Indigenous capability-based well-being. This contrasts with a previous study conducted in the UK by two of this study’s authors, in which a significant correlation was found between a capability-based well-being instrument (the GLiCS) for migrant women in high-income settings and the WHO-5 (Van der Boor et al., 2022).

The lack of significant correlations in the current study may also reflect differences in the constructs being measured along two main lines. The PHQ-9 focuses on depressive symptomatology and the WHO-5 focuses on indicators of well-being linked directly to health. These are interrelated domains (see, e.g. Topp et al., 2015) that both constitute specific dimensions of capabilities and functioning (low mood, feeling hopeless, etc.), rather than defining well-being more holistically (social relationships, attachment, etc.), as the EBK does. Second, the Kankuamo capabilities that emerged in the EBK are contextually situated, rather than reflecting dominant biomedical understandings of well-being (WHO-5) and depression (PHQ-9). We believe that such differences in contextual specificity can explain these findings of non-significance.

It may also be that the WHO-5 and the PHQ-9 lack adequate cultural fit for measuring well-being and mental health within the Kankuamo context. Indeed, making available interculturally developed assessment instruments for use within Kankuamo communities was a primary aim of the current research. Although both of these measures have been validated previously in other Indigenous contexts (Faruk et al., 2021; Williams et al., 2018), this does not necessarily mean they are appropriate for use in the Kankuamo context, or that a statistical validation process is the most contextually adequate. Previous concerns and criticisms have been voiced towards the use of western categories and measures of mental health in non-western settings (Snodgrass et al., 2017). Some authors argue that it is a basic error of validity and reliability to assume that because western mental phenomena can be identified in non-western settings, these mean the same as they do in the western world in terms of understandings, attributions and necessary remedies (Kirmayer & Pedersen, 2014; Summerfield, 2008). Instead, the use of western standardised tests might be open to issues of misinterpretation and cultural bias, with some authors considering whether attempts to ‘Indigenise’ standardised measures should be abandoned in favour of developing locally appropriate and culturally competent measures (Lindsey, 1998; McCabe, 2007; Robinson & Harris, 2005; Van Ommeren, 2003). For example, in a study of depression in Aboriginal men in central Australia, the authors found that the PHQ-9 required significant cross-cultural adaptation to be used in this setting (Brown et al., 2013). Thus, the findings that EBK measures are not significantly correlated with those of the WHO-5 and the PHQ-9 in the current study may reflect a more general inability of western standardised measures to speak to the unique cosmology of Kankuamo communities. These measures may not pick up on the importance of the mandate of the Law of Origin, which sets out the principles and elements that need to be respected to guarantee social co-existence, harmony and balance between all that exists. We acknowledge that the Law of Origin is directly referenced in only one item. However, its guiding philosophy is reflected across EBK items, illustrating the broad significance, in this context, of a cosmology that extends beyond the values reflected in purportedly universal measures of well-being and illness that were developed in settings dominated by western biomedical models. Unlike the universalising approaches of the WHO-5 and PHQ-9, the EBK does not stem from pre-existing categories of distress or illness. Instead, it was built inductively and interculturally based on qualitative data exploring local, self-reported capabilities based on ancestral knowledge that has been preserved by Kankuamo spiritual leaders (van der Boor et al., 2024). It also means, that the EBK measure should not be considered generalisable to other Indigenous contexts.

In the wider literature, there has been recognition of the need to harness culture-centred knowledge and perspectives from within Indigenous communities, to ensure that measures, interventions and programmes are relevant, effective and sustainable (Dickerson et al., 2020). Dickerson and colleagues (2020) published a review on encompassing cultural contexts within scientific research methodologies in the development of health promotion interventions. Their findings highlight the need to use community-based participatory approaches in which community members are involved from the outset and throughout the project, from initiation, to planning, implementation and evaluation, similarly to the TBPR. Such involvement is key to equitable and sustainable research relationships that can build community capacity and promote culture-centred interventions (Belone et al., 2017; Marina Apgar et al., 2016). Ultimately, these approaches can lead to outcomes more likely to decrease health disparities and enhanced positive outcomes of mental health and well-being (Dickerson et al., 2020).

A strength of the current study is that Kankuamo authorities and health leaders were involved from its conception, through design, implementation and validation, and held decision-making authority throughout. In addition, locally salient cosmologies and epistemologies were drawn upon in the analysis of qualitative data (van der Boor et al., 2024) to develop each of the EBK items. According to Wright and Lemmen (2012), this constitutes high levels of participation, and helps shift power relations away from those who traditionally define development priorities (governments, international organisations, high-income country researchers, etc.) towards Indigenous communities (Gigler, 2005). This type of approach is also well aligned with the CA and TBPR, because its open and participatory nature ensures that researchers’ values are not imposed, and seeks instead to build on local understandings and ways of doing (Fisher & Ball, 2003; Sen, 1989).

Given the high level of participation throughout this project, we believe that the EBK has achieved the aim of creating an intercultural measure of well-being and constitutes an important first step towards understanding the well-being of the Kankuamo people. For future research, it will be important to conduct a CFA of the EBK as a composite measure, as well as to consider additional item response theory analysis. The choice of research avenues to pursue should continue to be driven by the Kankuamo communities’ needs and views on how this measure, or items from within the measure, can best support the development of an intercultural health system that promotes the attainment of CA-based well-being within the Kankuamo communities. We also recommend running a known groups validity analysis (Davidson, 2014) to determine whether the EBK can differentiate between individuals identified by community informants as experiencing high levels of capability-based well-being versus those identified as experiencing low levels.

### Strengths, limitations and future directions

A key strength of this study is the aforementioned involvement of the Kankuamo community in generating a bottom-up approach to understanding well-being. An additional strength is the involvement of local community members in the research process. This strengthened local knowledge and capacity that can support future independent research within the community. Finally, the development of each of the EBK items was informed by qualitative data collected verbatim within the Kankuamo communities. A polychoric correlation analysis was used to validate the ordinal data, rather than the often erroneously used factor analyses developed for interval-level data (Kolenikov & Angeles, 2008).

Limitations include the possibility that the research process was influenced by acculturative pressures arising as the Kankuamo navigate the development of an intercultural health system that recognises their unique histories and knowledges, as well as their future aspirations. This process grapples with tensions between traditional and dominant epistemic and governance systems, which is an ongoing feature of the work of Kankuama IPS. Efforts to mitigate this tension include the use of a qualitatively driven approach, complemented by in-depth reflexive engagement and discussion, to developing a conceptual model of traditional knowledges and beliefs (Kankuama IPS, 2014). Moreover, the content of the EBK was designed through iterative discussion and agreement with community representatives and members. Another methodological limitation stems from the fact that community assessors collected data within their own communities, creating a risk of social desirability bias (Bergen & Labonté, 2020). Moreover, the study used a convenience sample, in which only 9 of 12 communities were represented. The remoteness of the remaining three entailed poor physical and digital access, making it impossible to collect data. Finally, the EBK is applied individually, which can create a bias towards individual well-being. This is a common critique of the CA's application in Indigenous settings (Gigler, 2005). Previous research suggests there is an urgent need to explore social and/or collective capabilities (e.g. spiritual rituals for community healing), as opposed to individual capabilities (e.g. access to local food), to reflect the worldviews of Indigenous peoples (Murphy, 2014). The methodology used to develop the EBK (focus groups, cognitive interviewing and consultation) allowed for the inclusion of items that speak to both individual and collective capabilities. An example of the first is item 10, which translates as ‘I have the freedom to make personal decisions that are important to me’, whereas the latter is illustrated in item 7, ‘Within my possibilities, I can contribute to the collective activities of the community’. Future research might explore how measures can be used in group settings, to measure group interactions and community levels of well-being. Another further research need highlighted by our findings is to assess the validity and reliability of western mental health measures within the Kankuamo communities. We recommend that workshops be conducted to gauge the cultural acceptability and understanding of the WHO-5 and PHQ-9, and for relevant adaptations to be made.

## Conclusion

This study, using a co-development approach, led to development of the EBK, the first assessment instrument for assessing capability-based well-being of the Kankuamo community. The different subscales of the EBK represent key individual and collective capability domains for the Kankuamo community. The findings highlight a need for caution in using western standardised mental health measures within the Kankuamo community, because these may lack adequate cultural fit. They also confirm the importance of listening to and working with local communities to identify their local priorities and needs. From a practical perspective, the EBK provides insight into well-being dimensions that are important for Kankuamo communities and can help inform guidelines for mental health and psychosocial support. It allows for capability measurement at the individual level, enabling the identification of items or sites of high capability, as well as those where support may be beneficial. It may also reveal capability differences among Kankuamo communities.

## Notes

1.
https://justiciaambientalcolombia.org/16027-2/

2. With the support of the universities of Liverpool, Belfast, Edinburgh and Externado de Colombia, with funding from the Colombian Ministry of Science and Technology and the United Kingdom Research Institute.

3. This one-year programme was made up of five participatory encounters with key actors for health within the territories (including health providers, health leaders, university students, etc.) to discuss and identify mental health and psychosocial needs in the Kankuamo territory. This programme was developed between the Externado University of Colombia, Kankuama IPS, and the Kankuamo authorities and health coordinators.

## Appendices Appendix A. Original Kankuamo Well-being Scale used for piloting V.01. Response options: strongly disagree, disagree, neutral, agree, strongly agree. Items in bold were retained in the final version of the EBK scale.

### Escala de Bienestar Kankuamo

1. **Tengo la oportunidad de orientar mis acciones a través de los principios de la Ley de Origen o mis propias creencias** (I have the opportunity to guide my actions through the principles of the Law of Origin or my own beliefs).

2. Tengo la oportunidad de vivir en mi territorio si lo deseo (I have the opportunity to live in my territory if I wish).

3. Puedo acceder a los espacios sagrados si lo deseo (I can access sacred spaces if I wish).

4. Puedo contribuir a la preservación de la cultura del pueblo Kankuamo (I can contribute to the preservation of the Kankuamo people's culture).

5. **Tengo la oportunidad de decidir entre usar medicina tradicional y occidental** (I have the opportunity to decide between using traditional and western medicine).

6. Soy capaz de vivir en armonía con la madre naturaleza (I am able to live in harmony with mother nature).

7. Tengo oportunidad de acceder a alimentos cultivados en mi territorio (I have the opportunity to access foods grown in my territory).

8. **Tengo oportunidad de producir (cultivar, criar) mis propios alimentos** (I have the opportunity to produce (grow, cultivate) my own food).

9. Tengo la oportunidad de acceder a recursos económicos que permiten atender las necesidades de mi hogar (I have the opportunity to access financial resources that allow me to meet my household's needs).

10. Mi salud física me permite hacer las actividades diarias que son importantes para mi (My physical health allows me to carry out the daily activities that are important to me).

11. Mi salud espiritual o mental me permite hacer las actividades diarias que son importantes para mi (My spiritual or mental health allows me to carry out the daily activities that are important to me).

12. Tengo la oportunidad de sentirme seguro en el territorio (I have the opportunity to feel safe in the territory).

13. Soy libre de participar en los distintos espacios de diálogo de mi comunidad (I am free to participate in the various spaces for dialogue within my community).

14. **Tengo la oportunidad de discutir mis intereses con las autoridades de mi comunidad** (I have the opportunity to discuss my interests with the authorities of my community).

15. **Dentro de mis posibilidades, puedo contribuir a las actividades colectivas de la comunidad** (Within my possibilities, I can contribute to the collective activities of the community).

16. **Puedo participar en actividades culturales y deportivas que son importantes para mi** (I am able to participate in cultural and sport activities that are important to me).

17. Soy capaz de cumplir con las funciones que tengo dentro de la comunidad (I am able to fulfil the functions that I have within the community).

18. Tengo la oportunidad de acceder a espacios educativos y de formación (I have the opportunity to access educational and training spaces).

19. Tengo la posibilidad de acceder a espacios o personas para aprender sobre saberes propios (I have the possibility to access spaces or people to learn about traditional knowledge).

20. **Tengo la libertad de tomar decisiones personales que son importantes para mi** (I have the freedom to make personal decisions that are important to me).

21. **Puedo expresar mi opinión libremente** (I am able to express my opinions freely).

22. Soy capaz de manejar mis emociones (I am able to manage my emotions).

23. **Soy capaz de mantener buenas relaciones con las personas que son importantes para mi** (I am able to maintain good relationships with the people that are important to me).

24. **Logro contribuir a la armonía en mi hogar** (I am able to contribute to the harmony within my home).

25. **Soy capaz de comportarme de acuerdo a las situaciones que se presentan** (I am able align my behaviour to different situations that occur).

26. Tengo la posibilidad de reflexionar sobre lo que pienso (I have the possibility to reflect on what I think).

27. **Soy capaz de reconocer y respetar a las personas por igual, independientemente de su género, orientación sexual, etc.** (I am able to recognise and respect people equally, regardless of their gender, ethnicity, age, relationship status, etc.).

28. **Dentro de mi territorio, me tratan con el mismo respeto y consideración, independientemente de mi género, orientación sexual, etc.** (Within my territory, I am treated with the same respect and consideration, regardless of my gender, ethnicity, age, relationship status, etc.).
